# Bad Effects of Vænesection in Cases of Poison from Opium

**Published:** 1814-10

**Authors:** John C. Yeatman

**Affiliations:** Member of the Royal College of Surgeons. Bristol, College Green


### *For the Medical and Physical Journal.*

### *Bad Effects of Venesection in Cases of Poison from Opium; by* Mr. John C. Yeatman, Surgeon, of Bristol.

BLEEDING is recommended and occasionally practised in the treatment of persons labouring under the sedative effects of an over-dose of opium; but, as I am acquainted with two cases which under the use of the lancet proved fatal, I am only performing a duty to society by recording them.

In the first case it will be seen that death immediately followed the sudden loss of blood from the temporal artery, although re-action had taken place: in the second, dissolution was the immediate consequence of opening the jugular vein whilst the patient was under the sedative influence of an over-dose of laudanum. It will be likewise seen, that the same plan of treatment which I so successfully adopted in the case of Mr. J. B., as narrated in your Journal for June, (vol. 31, page 468,) was pursued with the same striking advantage in one before us; and whilst it goes to condemn the early use of the lancet, it serves as an additional proof of the happy effects resulting from the employment of active emetics, vegetable acids, and powerful stimuli.

Case I.—In one of the spring months of the year 1798, my father was sent for, between the hours of eleven and twelve, p. m. to Mr. Myers, in Philadelphia, America, who had had swallowed, during the afternoon and evening, three drachms of opium. Mr. M. had been in the habit of keeping that drug in his pocket, and of biting off a morsel occasionally, "to allay passion," as he afterwards expressed himself, but had never before exceeded a few grains in the twenty-four hours. He was a middle-aged man, of the choleric temperament, and attempted to poison himself during a protracted altercation with his wife.

On visiting the patient, he was found in a state of insensibility; the eye-lids were closed, and the pupils dilated; the visage was pale and ghastly, and a clammy perspiration bedewed the forehead; the inferior maxilla was fixed to its fellow; the hands were clenched; the skin was cold; the breathing was slightly performed at long intervals, and the pulse small and intermitting.

The mouth being forced open, two scruples of the sulphat of zinc in solution were thrown into the stomach, and after irritating the fauces with a feather, a small quantity of its contents was discharged. A table-spoonful of mustard mixed in warm water, and sharpened with vinegar, vomited him freely, and the volatile alkali citric and acetic acids diluted were alternately administered. These means succeeded in restoring animation. The pulse began to beat more distinctly and regularly; a general warmth ensued; at length he was enabled to stand, and with assistance to walk up and down the room.

At eight o'clock, a. m. he spoke very rationally, was able to move about without help, and merely complained of slight headach, drowsiness, and weakness. A purgative was prescribed, and he was directed to drink plentifully of the vegetable acids and strong coffee.

In the course of the morning he appeared in every respect likely to do well—entered into a conversation on the enormity of attempting to destroy his life—and expressed much gratitude for the exertions that had been made towards his recovery.

My father again visited his patient in the afternoon, when, to his great astonishment, he found him on the bed in articulo mortis!—On inquiring the cause of this lamentable reverse, he was told that a physician, (through the interference of an officious friend, and without the knowledge of the patient,) had been sent for, who had taken nearly fourteen ounces of blood from the temporal artery. Mr. M. sunk under the operation, and died, as in a swoon, shortly afterwards.

Case II.—A man, æt. 40, admitted into hospital for some trivial complaint, was in a few days seized in a way which induced the medical gentlemen in attendance to consider sider him affected with apoplexy, and under that impression took sixteen ounces of blood from the jugular vein. This man suddenly sunk under the bleeding, and expired without a struggle immediately after. It was subsequently known, that he had been labouring under the effects of an over-dose of laudanum.

Bleeding has been thought proper in these cases, with a view to obviate congestion of blood in the brain and lungs. A celebrated physician, Dr. Cullen, has laid down the following "general directions" for their recovery; observing, however, that he cannot "treat this subject completely."

"When the poison taken into the stomach, or otherwise applied to the body, has already induced an apoplectic state, as those causes do commonly at the same time occasion a stagnation, or slower motion, of the blood in the vessels of the brain and of the lungs, so it will generally be proper to relieve this congestion, by taking some blood from the jugular vein, or from the veins of the arm." The doctor then goes on to observe: "Upon the supposition of a congestion in the brain or lungs, it will generally be proper to relieve it by means of acrid glysters, producing some evacuation from the intestines." And, also, "When these evacuations by blood-letting and purging have been made, the various stimulants which have been commonly proposed in other cases of apoplexy, may be employed here with more probability and safety," &c.

The principle being stated on which bleeding is founded in these cases, I will in the next place endeavour to ascertain in some degree the manner in which opium produces its effects in the animal system, and then proceed with great deference to suggest a few observations against the early use of the lancet. I need hardly observe, that it will be found extremely necessary to extract blood after the sedative effects of an over-dose of opium have been counteracted, should the balance of power be in favour of the heart and arteries.

The authors on the modus operandi of opium are numerous and celebrated; and the experiments and observations of Mead, Alston, Whytt, Fontana, Wilson, and others, are by far too extensive to enter largely upon them in this paper.

Mead imagines, that a poison may destroy life by acting on the nerves of the stomach and intestines, without being conveyed into the blood.

Alston conceives, that opium acts on the system in general, through the medium of the nerves to which it is applied.

Whytt says, "it remains, therefore, that opium, by affecting the extremities of the nerves of the part to which it is applied, does, by means of their connection and sympathy with the spinal marrow, destroy or prevent, through the whole nervous system, the operation of that power upon which depends sensation and motion in the bodies of animals."

Monro considers that opium, applied to the internal surface of the primæ viæ, operates more speedily, and in some cases more violently, through the nerves alone, than by absorption.

Cullen says, "the fumes rising from burning charcoal, the fumes of mercury, of lead, and of some other metallic substances; opium, alkohol,* and many other narcotic poisons; to all which I would add the power of cold, of concussion, of electricity, and of certain passions of the mind, produce apoplexy by directly destroying the mobility of the nervous power."

Concerning the influence of opium on the heart, Dr. Wilson has instituted some experiments to prove that that drug, applied to the coats of the blood-vessels, destroys their muscular power.† "Having adapted the web of a frog's foot to a microscope, I injected eight or ten drops of a solution (nearly as strong as the stronger solution) under the skin of the leg. In a few seconds the circulation became languid, and no motion could be perceived in some of the larger blood-vessels. It gradually became more obscure in the rest, till, in the space of about two or three minutes after the injection of the opium, it ceased altogether. Nor did this interruption of the circulation proceed from any general affection of the system, since the motion of the blood still continued in the other foot. This experiment was made three times in the same manner with the same result."—(For a further account of these experiments, see his Essay.)

Considering then that opium destroys the muscular power of the blood-vessels to which it is applied, it follows, when that drug is swallowed in large quantity, that the circulation of the blood in the branches and trunks of the right and left gastro-epiptoic arteries, the coronaria ventriculi, hepatica, splerica, superior and inferior mesenteric, will be impeded, and its return from the stomach, liver, spleen, pancreas, and

---

* Brodie has shewn by experiments on the different modes in which death is produced by certain vegetable poisons, (recorded in the Philosophical Transactions for the year 1811,) that *alkohol*, the essential oil of bitter almonds, the juice of the leaves of aconite, the empyreumatic oil of tobacco, and the woorara, produce death, by destroying the functions of the brain.

† "Opium, it was found, destroys the power of action in all the muscles to which it is immediately applied."—*Wilson's Essay on Opium.*

intestines, intestines, to the inferior cava, will be prevented, and, "one great source by which the heart is usually supplied with blood being in this manner suddenly cut off, it will send out a smaller quantity at each contraction; and, not being able, any more than the aorta and pulmonary arteries, immediately to adapt itself to the diminished quantity of blood, the latter will not be propelled with the same force as before, which will speedily retard, if not interrupt, the circulation, not only in the minute vessels of the extremities, (as we have seen exemplified in Dr. Alston's experiments,) but in the whole arterial system; the venous circulation will also be carried on in a very languid manner; the processes of secretion and excretion will cease almost universally; the skin will become cold, and lose its colour," &c.*

The narcotic and sedative effects of an over-dose of opium soon, and in some instances almost immediately, become manifest by giddiness, aversion to motion, dulness of the eyes, stupifaction, sleepiness, a loss of voluntary power, a cessation of the functions of the brain, an interruption to the performance of respiration, a languid and irregular action of the heart and arteries, coldness, convulsions, and death.

The energy of the nerves of the stomach and small intestines are greatly impaired or wholly destroyed by the extensive application of opium to those parts; and, through the medium of the eighth pair of nerves, the functions of the brain and power of the whole nervous system are suspended. Hence a state of insensibility, a loss of voluntary motion, an interruption to the performance of respiration,† universal coldness,‡ and a dilated pupil.

The small and scarcely perceptible pulsation of the heart and arteries, may be the consequence of the interruption of the circulation in the chylo-poietic and assistant chylo-poietic viscera. The check offered to the blood's passage through the lungs, and the deprivation of that quantum of nervous energy which is necessary to enable the heart and arteries to contract upon their contents, the stagnation of the blood in the vessels of the brain and of the lungs, will be the effect of a languid circulation, and the impediment given to its passage through the pulmonary vessels. Convulsions would

---

* Ward on Opium, Med. and Phys. Journal, vol. viii. p. 329.

† It has been also shewn, that the brain is directly necessary to the action of respiration.

‡ Mr. Brodie has proved by experiments (related in a paper read before the Royal Society, December 20, 1810,) the brain to be directly necessary to the generation of animal heat.

 appear appear to be the immediate effect of the opium, when it has been received into and conveyed by the blood to the brain.*

The question whether opium be a stimulant or a sedative, has long agitated the medical world; and even to this day the most eminent men differ on this subject. I am not at all disposed to enter the list of disputants, but I must observe, that the experiments and observations of most authors, and the

---

* Dr. Wilson perceived a solution of opium thrown into the heart pass along the aorta towards the brain, and he considered that convulsions arose from this circumstance.

The aorta was tied in twelve frogs, and, on throwing a strong solution of opium into the heart of each, that organ immediately ceased moving, and yet no convulsions supervened.

An experiment on ten frogs, in which the aorta was divided, was made with the same result.

The heart in six frogs was slit, the nerves remaining entire and uncompressed, and the heart still continuing to contract with vigour; when, on the application of a solution of opium to that organ, it instantly ceased moving, and yet no convulsions supervened.

In another experiment, "the aorta in four frogs was secured by ligature, and the auricle wounded so as to permit the blood to escape; in other two a ligature was thrown around all the vessels attached to the heart, and this organ was removed. The skull of each was then perforated; and after wounding the brain in the first four, a little of the weaker solution was dropped into it. In the other two, a few drops of a stronger solution were applied to the surface of the brain. In all of them the muscles of voluntary motion were seized with violent convulsions. They died with precisely the same symptoms which follow the injection of opium into the heart when it passes along the aorta."

Fontana, in his Treatise on Poisons, vol. i. p. 81, says, "That opium causes convulsions is owing, in my opinion, to its destroying at different times, and in an irregular way, the irritability of the muscular fibres. It is besides certain, that men and women of a delicate and weak frame, are always the most subject to convulsions; and it is not possible to suppose in these people a superabundance of animal spirits. We know that all the muscles, even in a relaxed state, preserve notwithstanding a certain tension of their fibres, which, when they are cut, never fail to contract themselves, and to enlarge the wound. When a muscle becomes paralytic, it lengthens, and its antagonist then contracts the more; which shows that the repose of the muscles depends on the equilibrium of strength betwixt the different muscles, and betwixt their different fibres. The powers thus balanced, destroy and renew themselves at every instant, without producing any motion or sensible change. This natural tension of the muscular fibres certainly depends on an equal and exact distribution of the fluids in the whole substance of the muscles. This truth is demonstrated the symptoms, as they occur in these cases, prove, notwithstanding men may differ on the properties of opium *in general*, that an over-dose* of that drug is a *direct sedative.*

It will be said, that, by drawing blood from the head, its vessels will be more equal to contract upon their contents, and congestion will be obviated; but in attempting this are we to deprive the body of power, whilst cold and almost incapable of performing a single function?

As opium does not appear to me to endanger the life of the patient by producing "a stagnation or congestion of the blood in the vessels of the brain and of the lungs," but kills by "destroying or preventing, through the whole nervous system, the operation of that power upon which depends sensation and motion in the bodies of animals;" so I con-

---

demonstrated in a dissertation which I published in the third volume of the Acts of Sienna, which was in part reprinted some time after at Lucca, with several considerable additions, and which was afterwards inserted in the first volume of my Animal Physics. But, if these muscles do not receive the same proportion of fluids, or if these fluids reach them, or are distributed amongst them with an unequal quickness and energy, the equilibrium of the mutual effort of the muscles is immediately destroyed; the strongest of them contract; and hence arise the convulsions and violent agitations of the whole frame. This is the reason why those who die of an hæmorrhage, as well as those who perish by poison, are seized with convulsions; for it certainly is not probable that the loss of blood and of strength should bear an equal proportion in every part, in every muscle, and in every fibre, whilst the circulation itself is unequal, and the muscular irritability is destroyed gradually, and in a very irregular way, according to time and circumstance."

* By an over-dose of opium, I mean a quantum capable of producing those effects which are enumerated in the cases of Mr. J. B., Mr. M., and others, and which take place *without any previous excitement.*

It is well known that a comparatively small dose of opium will accelerate the motion of the blood, and cause for a little while an increased flow of animal spirits. Hence it is generally to be seen at the toilet of the female of fashion; and hence the Turk, with his enervated frame, finds a temporary relief and pleasure in chewing it.

It is well known likewise, that a large quantity of opium may be taken without immediate injury, by persons who habituate themselves to its use. I saw a Turk under the piazzas of the great square of Valetta, in Malta, swallow half an ounce of that drug. I witnessed another on the Marina di Messina, in Sicily, take a still larger quantity; and I well recollect seeing a butcher at Baltimore, in America, drink three ounces of laudanum at a draught; observing, that it was impossible to perform his work without taking this his daily dram.

ceive ceive it will be first necessary to obviate this, by stimulating the nerves and destroying their energy. The cases of Mr. J. B., Mr. M., and others, show, that by exciting the action of the stomach by stimulating the muscular coats of its blood-vessels, and by rousing the energy of the nerves, *the functions of the brain and of the lungs* are restored, the blood being no longer impeded in its course, *congestion is obviated*, reaction is produced, general sensibility, warmth, and the voluntary powers return to their natural state, and the whole train of symptoms successively vanish. The hypotheses therefore founded on the supposed analogy between these cases and apoplexy, will be of little consequence while facts of the above description present themselves.

Again: if we be right in alleging that opium kills by its sedative influence *on the nerves of the primæ viæ, and through their medium by destroying the functions of the brain and the whole nervous system;* and that *the circulation is almost entirely prevented*, is not immediate bleeding very problematical? The sudden abstraction of blood, or the exhibition of any thing that will occasion *direct debility*, when the functions of the nervous and vascular systems are suspended or greatly interrupted, may deprive the body of the little power which remains, and which in this diminished state of the vis vitæ is easily destroyed.

Many practitioners consider bleeding proper, in the first instance, to obviate a determination of blood to the head, occasioned by the stimulating power of opium on the heart and arteries. A determination of blood to the head cannot, I think, take place in this manner. An over-dose of opium being a direct sedative, the heart and arteries are not invigorated but weakened, and the pulse soon becomes slightly perceptible to the touch and intermitting; whenever the above happens, it becomes apparent by an increased pulsation of the heart, carotid, and temporal arteries, an over-distension of the vessels of the neck, face, and scalp, and a redness and heat of the countenance and skin. The reverse of this is the case in a suspension of the vital powers from opium.

In the first stage of concussion of the brain, the symptoms are analogous to those of suspended animation from opium; as insensibility, loss of voluntary motion, a dilated pupil, a slow and hardly perceptible respiration, a weak and intermitting pulse, coldness of the skin, &c. Now, it is generally admitted, that the lancet will prove injurious in this stage of concussion. Mr. Hey, of Leeds, condemns bleeding "during the diminished state of the vis vitæ which immediately succeeds the injury."*

* Pract. Obs. in Surgery.

In

In cases of drowning, the interruption of the function of respiration, and of the action of the heart and arteries, will cause "a stagnation of the blood in the vessels of the brain and of the lungs;" and yet here, after inflating the lungs, we immediately endeavour to restore the balance of power by active internal and external stimuli. "Bleeding," says a practical writer (Dr. Thomas), "is a remedy which is often employed in cases of a suspension of the vital powers from drowning; but the natural heat should always be somewhat restored to the body, by a pursuance of the means which have been recommended, before we attempt to open a vein." And, in the following page, "while the body is cold, and the circulation and respiration are languid, I think blood-letting would be improper. If, however, after these functions and the natural temperature are restored, the patient should remain any time *in a comatose state, with a strong full pulse,* the propriety and necessity of venesection can hardly be doubted."

When a man falls from a great height, or receives a severe blow on the trunk, whereby a great shock is given to the nervous system, he is rendered insensible, the breathing becomes scarcely perceptible, and the pulse weak and intermitting, &c. The blood not being able to pass without great difficulty from the right ventricle to the left auricle of the heart, and a languid circulation, occasion congestion in the brain and lungs. It was formerly the common practice to bleed the apparently-lifeless sufferer on the ground where he received the injury, but the intelligent practitioner will now wait for reaction and a full pulse, before he deprives the patient of power, and in the first instance he will often think it right to administer a stimulant.

We find, then, that a person labouring under concussion, recovering from drowning, or from having sustained a severe shock, is not suffered to be bled until he is in some degree restored from the immediate effects of the injury or accident, although in all these instances "a stagnation or slower motion of the blood in the vessels of the brain and of the lungs" (for which bleeding is thought right in cases of suspended animation from opium) is in a more or less degree the consequence. From all that has been said, therefore, must we not infer—

1st, That congestion of blood in the brain and lungs, occurring as it does in persons labouring under the narcotic and sedative effects of opium, vanishes, as in the above cases, on the return of the vital functions, *without occasioning any previous danger?*

2dly, That bleeding in all *these* cases will prove fatal or dangerous dangerous if employed when the nervous and vascular systems are unable to perform their function?

The case of Mr. Myers, indeed, shows that bleeding may be fatal if adopted soon after re-action has taken place.

Numerous are the instances of recovery from the effects of of an over-dose of opium in which the lancet has not been used, but immediate recourse had to emetics and stimuli. Others again may be found in which internal stimuli alone proved successful.

Daniel Graham, æt. 35, swallowed two drachms and a hal. of opium, and was recovered by Mr. Sheppard, of New-York, by the exclusive exhibition of diluted brandy and wine. The opium had been in the stomach three hours before any thing was given him.*

I consider the cases of Mr. J. B. and Mr. M. striking instances of the dependance to be placed on the prompt and combined use of active emetics. Vegetable acids and powerful stimuli, and mustard, appears to be an excellent emetic and stimulant in these cases.

A very interesting case of apparent death from a large dose of opium, is related by Thomas Whately, Old Jewry, and communicated by the late Dr. William Hunter, in which recovery followed the repeated use of tartarized antimony, given to excite vomiting, and the inflation of the lungs.† On the other hand, I have known persons die from the effects of opium, when an emetic only has been administered.

John Danvers, æt. 60, swallowed six drachms of laudanum. This circumstance was not known for some hours, when an emetic of the sulphat of zinc was given, but without effect. He died before other means were tried. The next day I was present at the examination of the body. The vessels of the brain were full, without being in a state of over-distention. I did not discover any thing of consequence in the stomach and intestines, arising from the opium.

The treatment of these cases certainly forms an interesting and important subject. They do not very often occur, and rarely come within the limits of private practice. The comparative paucity of cases of this kind published cannot have escaped the notice of your numerous readers; and, if any thing herein noticed shall induce them to report those that have occurred to them, whether in favour of early bleeding or against it, much good will arise, and the little time I have taken in framing this imperfect communication will not have been lost.

---

* See Med. and Phys. Journal, vol. xix. p. 497.

† Med. Obs. and Inq. vol. vi. p. 331.

Before I conclude, I must admit that much may also be advanced on the other side of this important question, but nothing short of successful cases in illustration of early bleeding can possibly prove its utility, or place it in a less equivocal point of view. Such cases should be well attested before hypotheses, however specious they may appear, should induce us to bleed our patient while labouring under the sedative effects of an immoderate dose of opium.

JOHN C. YEATMAN,

*Member of the Royal College of Surgeons.*

*Bristol, College Green, August* 10, 1814.

